# The NLRP3 Inflammasome in Non-Alcoholic Fatty Liver Disease and Steatohepatitis: Therapeutic Targets and Treatment

**DOI:** 10.3389/fphar.2022.780496

**Published:** 2022-03-08

**Authors:** Lili Yu, Wei Hong, Shen Lu, Yanrong Li, Yaya Guan, Xiaogang Weng, Zhiwei Feng

**Affiliations:** ^1^ School of Basic Medical Sciences, Xinxiang Medical University, Xinxiang, China; ^2^ Institute of Precision Medicine, Xinxiang Medical University, Xinxiang, China; ^3^ The Third Clinical College of Xinxiang Medical University, Xinxiang, China; ^4^ Xinxiang Key Laboratory of Tumor Vaccine and Immunotherapy, Xinxiang Medical University, Xinxiang, China

**Keywords:** NLRP3, inflammasome, NAFLD, NASH, therapeutic agents

## Abstract

Non-alcoholic fatty liver disease (NAFLD) is among the most prevalent primary liver diseases worldwide and can develop into various conditions, ranging from simple steatosis, through non-alcoholic steatohepatitis (NASH), to fibrosis, and eventually cirrhosis and hepatocellular carcinoma. Nevertheless, there is no effective treatment for NAFLD due to the complicated etiology. Recently, activation of the NLPR3 inflammasome has been demonstrated to be a contributing factor in the development of NAFLD, particularly as a modulator of progression from initial hepatic steatosis to NASH. NLRP3 inflammasome, as a caspase-1 activation platform, is critical for processing key pro-inflammatory cytokines and pyroptosis. Various stimuli involved in NAFLD can activate the NLRP3 inflammasome, depending on the diverse cellular stresses that they cause. NLRP3 inflammasome-related inhibitors and agents for NAFLD treatment have been tested and demonstrated positive effects in experimental models. Meanwhile, some drugs have been applied in clinical studies, supporting this therapeutic approach. In this review, we discuss the activation, biological functions, and treatment targeting the NLRP3 inflammasome in the context of NAFLD progression. Specifically, we focus on the different types of therapeutic agents that can inhibit the NLRP3 inflammasome and summarize their pharmacological effectiveness for NAFLD treatment.

## Introduction

Non-alcoholic fatty liver disease (NAFLD) is among the most common chronic liver diseases globally and is defined by hepatic steatosis (hepatic triglyceride by liver weight >5%) without significant alcohol consumption. The incidence of NAFLD is increasing, with a prevalence of approximately 20–30% of populations worldwide and 75–100% in individuals with obesity (Dewidar et al., 2020; Scorletti and Carr, 2021). The progressive form of NAFLD is non-alcoholic steatohepatitis (NASH), which combines steatosis with inflammation and fibrosis, and is the leading cause of end-stage liver disease. Unlike simple steatosis, NASH is irreversible and can eventually progress to fibrosis, cirrhosis, or even hepatocellular carcinoma (HCC). Approximately 10–25% patients with NASH may be at risk of progression to cirrhosis according to current estimates (Friedman et al., 2018). Moreover, cirrhosis is a leading cause and risk factor for liver transplantation.

The pathogenesis of NAFLD is not fully understood. The concept of “multiple hits” has been proposed and is currently the most accepted explanation for NAFLD pathogenesis. Cross-talk and interactions between host genetics and environmental factors are characterized as the occurrence of parallel multiple hits. Among the most important hits is lipid metabolism disorder, characterized by increased triglyceride accumulation and excess lipid deposition in hepatocytes (Dewidar et al., 2020), which is the key step for the development of isolated steatosis. Other hits in hepatocytes, such as lipid peroxidation, mitochondrial dysfunction, and excessive reactive oxygen species (ROS) production during oxidative stress, promote inflammation, cell death, and progressive liver damage. These hits activate inflammatory cascades and pro-inflammatory cytokines, including interleukin (IL)-1*β*, tumor necrosis factor-*α* (TNF-*α*), and IL-6, which contribute to NAFLD pathogenesis and cause subsequent progression to fibrogenesis (Cortez-Pinto et al., 2006), eventually leading to an increase in the prevalence of NAFLD/NASH. Furthermore, if there is no effective treatment, the disease will continue to develop into liver fibrosis, cirrhosis, and even HCC. Despite extensive research into NAFLD, the detailed mechanisms underlying this condition remain unclear, and there are no effective pharmacological agents for its treatment. Therefore, development of effective treatments is required.

Inflammasomes contribute to the pathogenesis of various chronic and acute liver diseases (Szabo and Petrasek, 2015; Yu L. et al., 2019). Inflammasomes are complexes formed by multiple intracellular proteins, which can detect damage-associated molecular patterns (DAMPs) generated by damaged cells and pathogen-associated molecular patterns (PAMPs) from gut–liver axis pathogens. The assembly of these complexes induces the activation of caspase-1, which proteolytically induces the maturation of cytokines pro-IL-1*β* and pro-IL-18 to IL-1*β* and IL-18, respectively. Furthermore, this process induces pore formation on cell by gasdermin D (GSDMD) and stimulates pyroptosis and release of inflammatory factors into the extracellular space. Various inflammasome components, such as the nucleotide-binding domain and leucine-rich repeat family pyrin domain-containing proteins (NLRP1, NLRP2, NLRP3, NLRP6, NLRP10, and NLRP12), have been identified and found to play important roles in various experimental models and human liver diseases (Szabo and Petrasek, 2015; Mohamadi et al., 2018). Although other types of inflammasome are also related to liver diseases, NLRP3-containing inflammasome has been widely studied in this context, and multiple studies have demonstrated that NLRP3 inflammasome is a key contributor to amplification of hepatic inflammation, immune cell activation, and hepatocyte damage (Szabo and Petrasek, 2015; Swanson et al., 2019).

NLRP3 inflammasome is essential for processing the main pro-inflammatory cytokines, which represent a new target for the prevention and treatment of NAFLD/NASH. Recently, findings related to NLRP3 inflammasome-associated agents and inhibitors for use in treating NAFLD/NASH in experimental models have supported this therapeutic approach and been demonstrated to have positive effects. The translation and implementation of these findings into therapeutic support could be a novel approach for the treatment of inflammation-induced liver disease. In this review, we discuss the activation, biological functions, and treatments targeting NLRP3 inflammasomes in the context of NAFLD progression. Specifically, we focus on the different types of therapeutic agents available for the inhibition of the NLRP3 inflammasome and summarize pharmacological treatments for NAFLD involving NLRP3 blockade.

## Activation of the NLRP3 Inflammasome in Non-Alcoholic Fatty Liver Disease/Non-Alcoholic Steatohepatitis

The NLRP3 inflammasome is essential for the activation of caspase-1, and consequently, the processing of key pro-inflammatory cytokines and pyroptosis; all of these processes are implicated in the development of NAFLD. The assembly of the NLRP3 inflammasome requires interaction among the NLRP3 receptor, the apoptosis-associated speck-like protein (ASC) adapter, and pro-caspase-1, which depends on binding *via* specific protein domains. There are three conserved domains in the NLRP3 receptor, a nucleotide-binding and oligomerization (NACHT) domain in the mid-part of the protein, a leucine-rich repeat domain at the C-terminus, and a pyrin domain (PYD) at the N-terminus. The ASC adapter also has a C-terminal PYD domain, as well as a large protein–protein interaction motif, the caspase activation recruitment domain (CARD), at its N-terminal. When the NLRP3 inflammasome assembles, NLRP3 acts as a scaffold *via* its NACHT domain, ASC oligomerizes through the interaction between ASC and NLRP3 through the PYD domains, and pro-caspase-1 is activated through a homophilic interaction of the CARD with ASC (Mariathasan et al., 2004).

**FIGURE 1:**
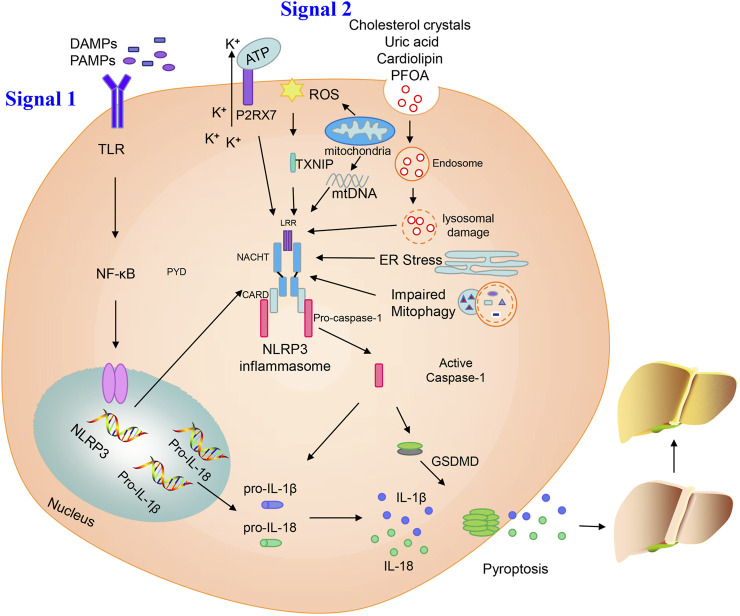
Activation of the NLRP3 inflammasome. Priming and activation of the NLRP3 inflammasome involve two steps. First, inflammasome activation is triggered by priming signals, including various DAMPs and PAMPs, which can lead to NF-κB-mediated upregulation and expression of NLRP3, pro-IL-18, and pro-IL-1*β*. The second step is inflammasome formation, which is triggered by specific stimuli; the stimuli involved are diverse and depend on cellular stresses during NAFLD/NASH, and include extracellular ATP, mitochondrial ROS, mtDNA, cholesterol crystals, uric acid, cardiolipin, PFOA, ER stress, and impaired mitophagy. NLRP3 inflammasome stimulation can lead to self-cleavage of pro-caspase-1 to generate the active form, caspase-1, which subsequently cleaves pro-IL-18 and pro-IL-1*β* to generate their mature forms, L-18 and IL-1*β*, for secretion. Activated caspase-1 can also induce GSDMD-mediated pyroptosis. Finally, NLPR3 inflammasome activation can instigate a series of serious conditions, ranging from benign NAFLD to fibrosis, and ultimately cirrhosis and hepatocellular carcinoma.

The NLRP3 inflammasome has a two-step initiation and activation model (Figure 1). The first step in inflammasome induction is initiation of the priming signal, primarily comprising DAMPs and PAMPs, for example, high mobility group box 1 (HMGB1) or lipopolysaccharide (LPS), leading to the activation and upregulation of NLRP3, pro-IL-1*β*, and pro-IL-18; this is the key step mediating NLPR3 expression. The second step is triggering of inflammasome formation by specific stimuli, which is the signal for caspase-1 activation. The NLRP3 inflammasome mediates pro-caspase-1 self-cleavage to generate active caspase-1, which facilitates generation of the secretory molecules, IL-1*β* and IL-18, from pro-IL-1*β* and pro-IL-18, respectively. Caspase-1 can also induce GSDMD expression, which mediates plasma membrane pore formation and osmotic swelling of the cell, as well as inducing pyroptosis, leading to a pro-inflammatory cytokine release cascade (Wree et al., 2014).

Various cellular stress-dependent signals mediate the second step of NLRP3 inflammasome activation. Given the structural and chemical diversity of stimulators, NLRP3 cannot physically interact with all of these activators. In NAFLD, various stimuli can activate the NLRP3 inflammasome, such as extracellular adenosine triphosphate (ATP), cholesterol crystals, mitochondrial ROS, mitochondrial DNA (mtDNA), uric acid, cardiolipin, perfluorooctanoic acid (PFOA), endoplasmic reticulum (ER) stress, and impaired mitophagy (Figure 1).

Extracellular ATP acts as an internal danger signal and directly activates plasma channels *via* P2X purinergic receptor 7 (P2RX7), resulting in intracellular influx of K^+^, which is an important direct trigger of NLRP3 inflammasome activation. Deletion of the *P2RX7* gene can protect mice from severe symptoms in a model of high-fat diet (HFD)-induced NASH, likely by blocking NLRP3 inflammasome activation (Blasetti Fantauzzi et al., 2017). Baeza-Raja et al. (2020) also indicated that P2RX7 inhibitor treatment can ameliorate the histological features of NASH, improving liver fibrosis and inflammation.

ROS has been proposed as a common signal for NLRP3 inflammasome activation, which has long been considered to cause lethal hepatocyte injury during the process of steatosis. Increased ROS generation, induced by elevated free fatty acid content, enhances lipotoxicity within steatotic hepatocytes (Marra and Svegliati-Baroni, 2018). Zhang et al. (2015) showed that the ROS–thioredoxin interacting protein (TXNIP) pathway mediated hepatocellular NLRP3 inflammasome activation in a fructose-induced NAFLD model. Moreover, inhibition of the TXNIP-NLRP3 axis can restore intestinal barrier function *via* repression of ROS-mediated oxidative stress in a NASH model (Bai et al., 2019). Mitochondria are another source of ROS *via* their respiratory function. Dysfunctional mitochondria-induced pyroptosis and the subsequent production of ROS contribute to NAFLD development. NLRP3 inflammasome triggered by impaired mitophagy is the key point in progression from NAFLD to NASH (Zhang et al., 2019). In a mouse model of methionine/choline-deficient (MCD) diet-mediated steatohepatitis, control of ROS by Tim3, a promising protective factor, can alleviate the pathological features and associated NLRP3 inflammasome activation in macrophages (Du et al., 2019). ROS generation and stimulation of NLRP3 signaling can also be inhibited by X-linked inhibitor of apoptosis protein (XIAP) in a model of HFD-induced hepatic steatosis, which may be an indispensable apoptosis regulator (Zilu et al., 2019).

Cytosolic oxidized mtDNA has long been considered a trigger for NLRP3 inflammasome activation. Injured mitochondria can release fragmented mtDNA, facilitating conversion of mtDNA to an oxidized form, which acts as the final NLRP3 activator (Shimada et al., 2012). Cytidine/uridine monophosphate kinase (CMPK2)-dependent mtDNA synthesis is required for the production of oxidized mtDNA fragments, allowing for exposure to NLRP3 activation (Zhong et al., 2018). In a mouse model of NASH, mtDNA released from mitochondria on fatty acid stimulation can activate the NLRP3 inflammasome and aggravate NASH development (Pan et al., 2018).

Cholesterol crystals act as endogenous danger signals, which can activate the NLRP3 inflammasome (Duewell et al., 2010). Many cholesterol crystals are present in hepatocyte lipid droplets, particularly within the steatotic liver of patients with NASH, and their accumulation induces lipotoxicity in hepatocytes, promoting NASH development (Van Rooyen et al., 2011; Farrell et al., 2018). In mouse models of NASH induced by a high-fat, high-cholesterol diet, exposure of Kupffer cells, hepatocytes, and bone marrow macrophages to cholesterol crystals stimulates NLRP3 inflammasome activation and mediates NASH development (Ioannou et al., 2015; Mridha et al., 2017).

Uric acid can directly induce hepatocyte fat accumulation and hepatic steatosis *via* the NLRP3 inflammasome. Levels of serum uric acid are significantly increased in patients with NAFLD and independently predict an increased incidence of NAFLD in healthy individuals. Serum uric acid lowering therapy by using allopurinol inhibits NLRP3 inflammasome activation in a HFD model of NAFLD (Wan et al., 2016). Furthermore, atorvastatin reduces levels of uric acid, ameliorating NAFLD/NASH and preventing liver fibrosis (Paschos et al., 2018). Uric acid production is catalyzed by the rate-limiting enzyme, xanthine oxidase, which can regulate the activation of the NLRP3 inflammasome (Xu et al., 2015).

Cardiolipin is an anionic phospholipid that contains four acyl chains and two phosphatidyl groups linked to a glycerol backbone. Cardiolipin is effective in stimulating NLRP3 inflammasome through a ROS-independent signaling pathway, and its deficiency is protected against hepatic steatosis (Peng et al., 2018). Upregulation and activation of the NLRP3 inflammasome by cardiolipin is crucial in NASH pathogenesis. Moreover, the inhibition of cardiolipin can suppress NLRP3 inflammasome activation, thereby improving and ameliorating symptoms of NASH (Liu et al., 2019).

Perfluorooctanoic acid (PFOA) is an industrial waste product that persists in the environment and is used in many industrial processes. Exposure to PFOA can increase NLRP3 aggregation and enhance IL-1*β* production, leading to the development of NAFLD. Autophagy is also involved in hepatic NLRP3 inflammasome activation and lipid metabolism disorder following PFOA treatment (Weng et al., 2020).

ER stress is a protective process that restores protein homeostasis by activating the unfolded protein response. It has also been proposed that NLRP3 inflammasome activation in hepatocytes can be induced by ER stress, potentially leading to liver damage (Lebeaupin et al., 2015). ER stress-triggered hepatic NLRP3 inflammasome formation can be prevented by farnesoid X receptor (FXR) activation, which downregulates ER stress-mediated protein kinase R-like ER kinase (PERK) activation, thereby ameliorating liver injury (Han C. Y. et al., 2018).

Mitophagy influences NLRP3 inflammasome activation in hepatic lipotoxicity. In a murine model of NASH, hepatic NLRP3 inflammasome formation can be triggered by impaired mitophagy. Furthermore, damaged mitophagy in primary hepatocytes leads to successive NLRP3 inflammasome stimulation, which in turn causes inflammation and progression from NAFLD to NASH (Zhang et al., 2019).

## Role of the NLRP3 Inflammasome in Non-Alcoholic Fatty Liver Disease/Non-Alcoholic Steatohepatitis Models

A number of studies have focused on the role of the NLRP3 inflammasome in NAFLD progression and indicated its essential status. NLRP3 is primarily and highly expressed in macrophages, hepatic stellate cells (HSCs), and hepatocytes, and its activation greatly aggravates NAFLD and secretion of pro-inflammatory cytokines, IL-1*β* and IL-18 (Swanson et al., 2019). Levels of inflammasome components, including NLRP3, caspase-1, pro-IL-1*β*, and pro-IL-18, are significantly higher in liver samples from patients with NASH than in those without the disease, and their expression is associated with the development/degree of fibrosis (Csak et al., 2011; Wree et al., 2014). Caspase-1, the activated form after inflammasome activation, is also present in the serum of patients with NAFLD, and its levels are closely correlated with disease severity (Gaul et al., 2021).

Consistent with these results, experimental studies have demonstrated direct roles for NLRP3 inflammasomes in mouse models during NAFLD/NASH development. Cai et al. (2017) showed that NASH development was blocked in NLRP3 knockout mice, as well as alleviated palmitic acid-induced inflammation in Kupffer cells. Similarly, in another HFD-induced NAFLD mouse model, histological evidence suggested that compared to wild-type mice, NLRP3 ablation resulted in reduced hepatic steatosis (Vandanmagsar et al., 2011). Gaul et al. (2021) also showed that the loss of NLRP3 could protect from infiltration of activated macrophages, liver injury, diet-induced steatohepatitis, and fibrosis. Gallego et al. (2020) indicated that the absence of NLRP3 can regulate the liver fibrosis process and attenuate age-related liver fibrotic pathology during aging. However, knockin mice with NLRP3 exhibited spontaneous liver fibrosis, even when fed normal chow diet. Furthermore, when treated with low dose of LPS, these mice showed increase in sensitivity to inflammation and liver damage (Gaul et al., 2021). Temporal overexpression of NLRP3 in mice can lead to earlier and more serious pathological features of diet-induced steatohepatitis in another NLRP3 knocking mouse model (Wree et al., 2014). In contrast, only a few studies have indicated that NLRP3 negatively modulates NAFLD/NASH progression *via* regulation of gut microbiota (Henao-Mejia et al., 2012). The lack of NLRP3 caused a worse phenotype in an NAFLD mouse model, with disruption of the gut–liver axis and gut dysbiosis (Pierantonelli et al., 2017).

In addition to NLRP3, experimental studies have demonstrated that caspase-1 is also involved in NAFLD/NASH (Dixon et al., 2012), as it can process pro-IL-1*β* to its bioactive form, IL-1*β*, which has a key role in accelerating NAFLD development and inflammation induced by obesity (Mirea et al., 2020). Mirea et al. (2020) showed that caspase-1 deficiency ameliorated diet-induced weight gain, adipose tissue inflammation, and liver steatosis. Another group also demonstrated that mice deficient of caspase-1 were protected from HFD or MCD diet-mediated early fibrogenesis, hepatic steatosis, and inflammation (Dixon et al., 2012; Dixon et al., 2013). Furthermore, other studies have indicated that caspases-1/11 have active roles in NAFLD by modulating the composition and diversity of the gut microbial community and regulating liver lipid composition and metabolism (de Sant'Ana et al., 2019). Inhibition of caspase-1 can modulate the capability of this enzyme and attenuate NASH development. The administration of Ac-YVAD-cmk, a caspase-1 inhibitor, can ameliorate insulin resistance, improve liver fibrosis progression, and delay NASH development in male low-density lipoprotein receptor (LDLR) knockout Leiden mice.

Pyroptosis is characterized by lytic cell death and inflammatory factor release, due to NLRP3 inflammasome activation and pro-inflammatory cytokine cascade, and promotion of cell lysis through formation of gasdermin D (GSDMD) protein pores (Wu et al., 2019). GSDMD is the executive molecule in pyroptosis, and N-terminal fragments of GSDMD (GSDMD-N), which induce pyroptosis, are markedly upregulated in the livers of patients with NAFLD/NASH (Xu et al., 2018), and are essential in the pathogenesis of steatohepatitis, through regulation of cytokine secretion, control of nuclear factor-κB (NF-κB) activation, and inhibition of lipogenesis. Deficiency of GSDMD in mice typically leads to relief of the NASH phenotype, including reduction of inflammation, attenuation of steatosis, and amelioration of fibrosis, while overexpression of GSDMD augments liver inflammation and steatosis following feeding of an MCD diet (Xu et al., 2018). Moreover, after activation of NLRP3 inflammasome, hepatocytes can undergo pyroptosis, with subsequent release of NLRP3 inflammasome particles that stimulate stellate cell activation and amplify liver fibrosis. Hepatic stellate cells can engulf these released NLRP3 inflammasome particles to cause fibrosis, and release of these particles can be blocked by inhibitor of endocytosis, cytochalasin B (Gaul et al., 2021).

## Pharmacological Agents Associated With NLRP3 Inflammasome Suppression for Non-Alcoholic Fatty Liver Disease/Non-Alcoholic Steatohepatitis Treatment

**FIGURE 2:**
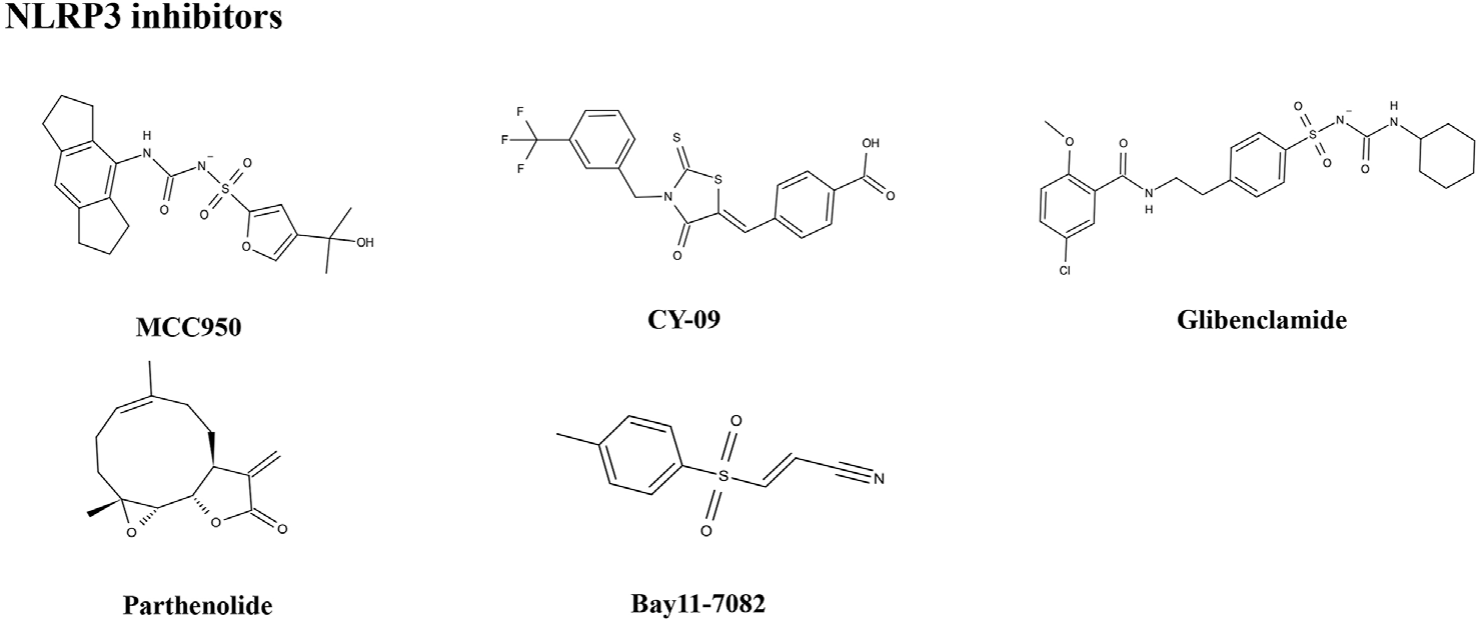
Structures of NLRP3 inhibitors.

**FIGURE 3:**
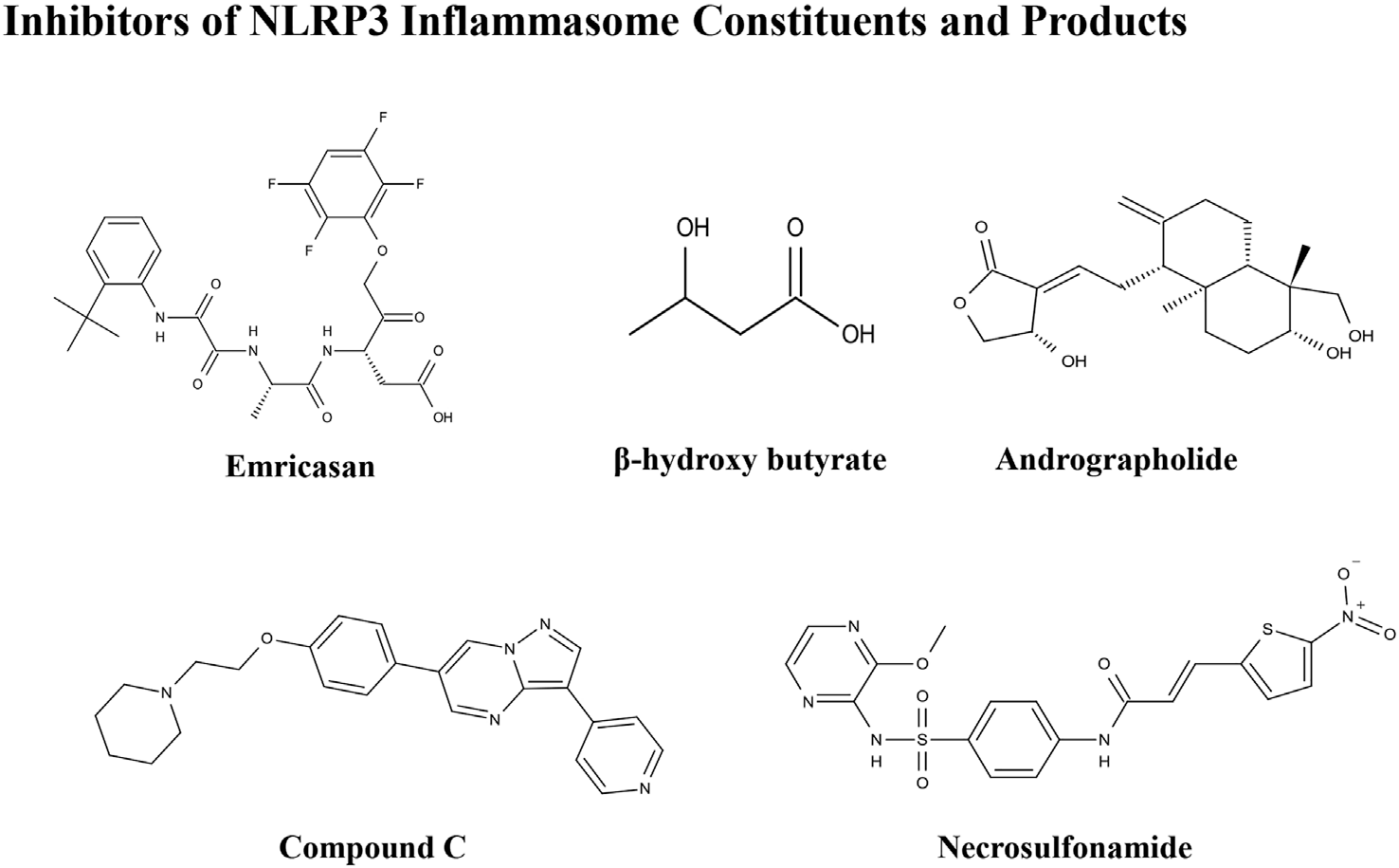
Structures of NLRP3 inhibitors of inflammasome constituents and products.

**FIGURE 4:**
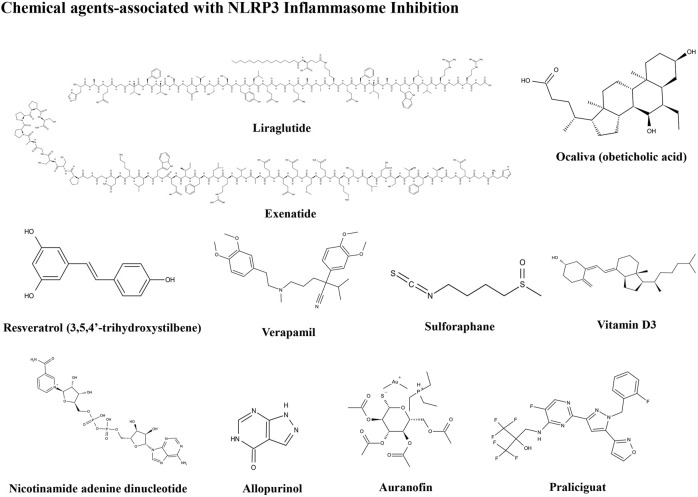
Structures of chemical agents.

**FIGURE 5:**
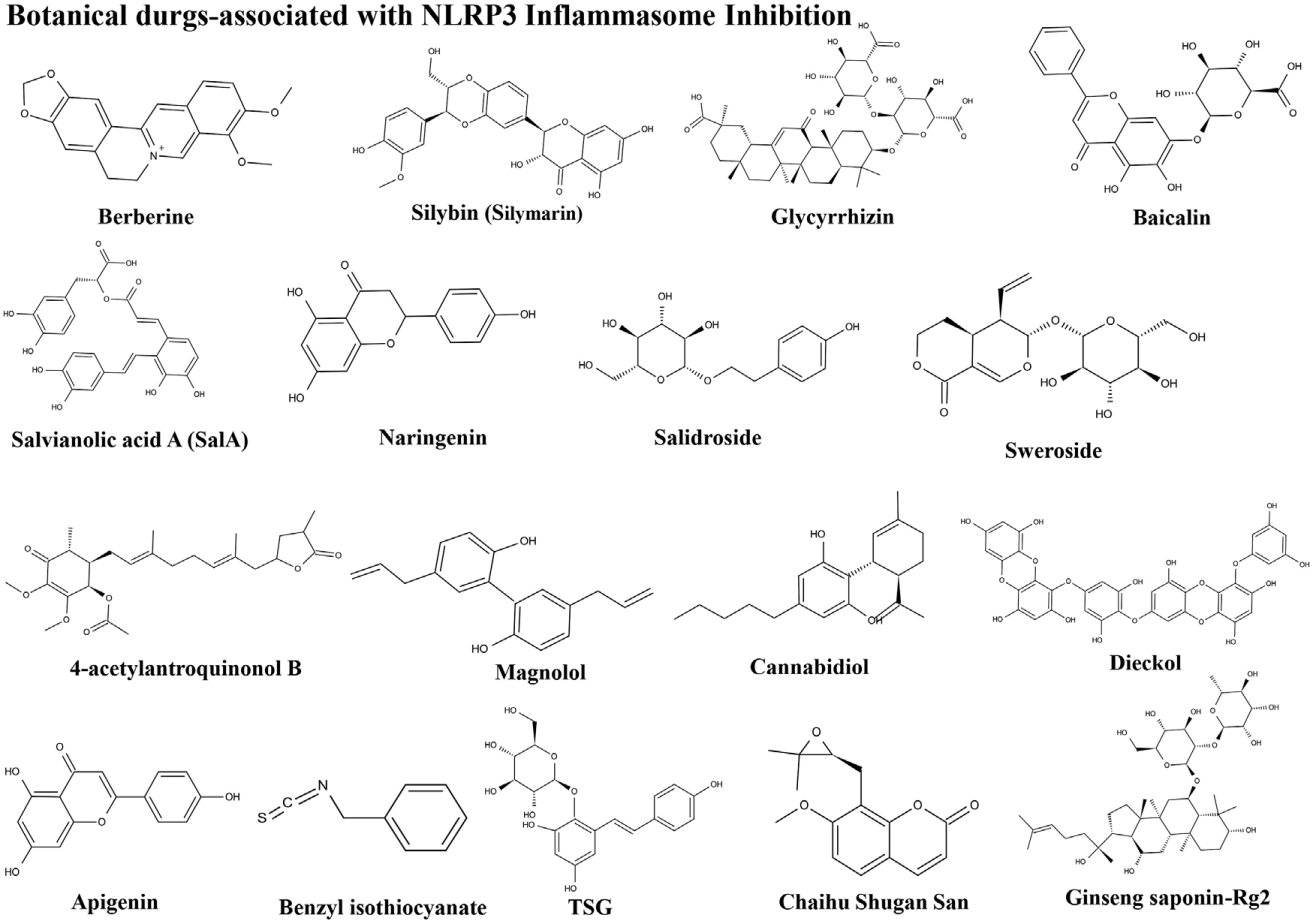
Structures of herbal agents.

The importance of the NLRP3 inflammasome in NAFLD/NASH progression demonstrates the feasibility of intervention with therapeutic agents targeting this process, which is urgent for the treatment of this disease (Swanson et al., 2019). Given the complexity of NLRP3 inflammasome signaling cascades, activation of various target proteins can be suppressed in many ways, including blockade of upstream signals, inhibition of inflammasome assembly, suppression of caspase-1 activation, prevention of GSDMD cleavage, and arrest of inflammatory cytokines production (Zahid et al., 2019). Various strategies to target the NLRP3 inflammasome have been developed, including NLRP3 inhibitors targeting the NLRP3 receptor (Table 1), inhibitors targeting constituents and products of the NLRP3 inflammasome (Table 2), chemical agents associated with NLRP3 inflammasome blockade (Table 3), and traditional botanical drugs related to NLRP3 inflammasome inhibition (Table 4). In the following text, we describe recently developed pharmacological agents associated with the NLRP3 inflammasome signaling pathway and propose their potential therapeutic mechanisms in the treatment of NAFLD/NASH. The chemical structures of these ligands are present in Figure 2 (NLRP3 inhibitor), Figure 3 (inhibitors of NLRP3 constituents and products), Figure 4 (chemical agents associated with NLRP3 inflammasome inhibition), and Figure 5 (botanical drugs associated with NLRP3 inhibition).

**TABLE 1:** NLRP3 inhibitors.

Inhibitor	Inhibition mechanism	Specificity	Inhibition of priming	Clinical status	Refs.
MCC950	Binds Walker B motif; NACHT ATPase inhibitor	NLRP3	No	Phase 2	Zahid et al. (2019)
CY-09	Binds Walker A motif; NACHT ATPase inhibitor	NLRP3	No	—	Jiang et al. (2017)
Glibenclamide	Inhibits ATP-sensitive K^+^ channels	NLRP3	No	Phase 4	Dwivedi and Jena, (2020)
Parthenolide	NACHT ATPase inhibitor and caspase-1 inhibitor	NLRP3	Yes	—	Bahabadi et al. (2017)
		AIM2			
		NLRC4			
		NLRP1			
Bay11-7082	NACHT ATPase inhibitor	NLRP3	Yes	—	Jiang et al. (2017), Yongsheng Yu et al. (2019)
		NLRC4			

**TABLE 2:** NLRP3 constituents inhibitors.

Inhibitor	Inhibition mechanism	Clinical status	Refs.
Emricasan	Caspase inhibitor	Phase 3	Barreyro et al. (2015), Garcia-Tsao et al. (2020)
*β*-Hydroxybutyrate	Inhibits K^+^ efflux; inhibits ASC speck oligomerization	—	Youm et al. (2015)
Andrographolide	NF-κB inhibitor	—	Cabrera et al. (2017)
Compound C	ATP-competitive AMPK inhibitor	—	Wang et al. (2019)
Necrosulfonamide	GSDMD inhibitor	Phase 2	Majdi et al. (2020)
SGM-1019	P2X7 receptor inhibitor	Phase 2	Di Virgilio et al. (2017), Alharthi et al. (2020)

**TABLE 3:** Chemical agents.

Chemical medicines associated with NLRP3 inflammasome suppression		
Liraglutide	Exenatide	Ocaliva
Resveratrol	Verapamil	Vitamin D
NAD	Allopurinol	Auranofin
Praliciguat	—	—

**TABLE 4:** Traditional botanical drugs.

Botanical drug associated with NLRP3 inflammasome suppression		
Berberine	Silybin	Glycyrrhizin
Baicalin	LBP	Salvianolic acid A
Naringenin	Salidroside	Ginseng saponin
Sweroside	4-AAQB	Magnolol
Cannabidiol	Dieckol	Apigenin
*Antrodia cinnamomea*	Benzyl isothiocyanate	TSG
Sulforaphane	Chaihu Shugan San	Fufang zhenzhu tiaozhi
Gegen Qinlian Decoction	—	—

### NLRP3 Inhibitors

#### MCC950

The small molecule, MCC950, is a diarylsulfonylurea-containing compound that was the first small molecule identified as an NLRP3 inhibitor. MCC950 blocks the activation of NLRP3 inflammasomes and IL-1*β* production by preventing ASC oligomerization. MCC950 can interact directly with Walker B motif in the NACHT domain of NLRP3, abrogating ATP hydrolysis and thereby suppressing NLRP3 activation and inflammasome formation (Coll et al., 2019). Treatment with MCC950 has potential to reduce liver inflammation and fibrosis, improving NAFLD pathology (Mridha et al., 2017). Furthermore, pretreatment with MCC950 in macrophages can reverse inflammasome activation and pyroptotic cascade (Zhang et al., 2019). In addition, MCC950 abolished the upregulation of NLRP3 expression, stimulation of caspase-1, and secretion of IL-1*β* induced by an atherogenic diet. In an MCD diet model of NASH, MCC950 reduced liver inflammation and protected mice from hepatic fibrosis, with no negative effect on metabolic risk or steatosis factors (Mridha et al., 2017). A clinical trial to evaluate the safety and efficacy of NAFLD/NASH treatment with MCC950 is urgently needed.

#### CY-09

CY-09 significantly inhibits NLRP3 inflammasome formation, both *in vivo* in mouse models and *ex vivo* in human cells, and was selected as a direct and effective NLRP3 inhibitor (Jiang et al., 2017). CY-09 can directly interact with the NLRP3 Walker A motif to prevent ATP binding to NLRP3. Caspase-1 activation and IL-1*β* release caused by monosodium urate, ATP, and nigericin-induced NLRP3 stimulation are dose-dependently suppressed by CY-09 in bone marrow-derived macrophages. In a HFD-induced mouse model, CY-09 reduced hepatic steatosis in experimental NAFLD mice (Wang X. et al., 2021). In another mouse model, CY-09 combined with endoscopic sleeve gastroplasty reduced body weight, improved insulin resistance, and alleviated hepatic steatosis (Sun K. et al., 2020). Similar to MCC950, there have been no clinical studies reported regarding use of CY-09 for the treatment of NAFLD/NASH, and further investigations are warranted.

#### Glibenclamide

The sulfonylurea drug, glibenclamide, is extensively administered for the treatment of T2DM. The mechanism of action of glibenclamide involves inhibition of ATP-sensitive K^+^ channels, making it a potent inhibitor of NLRP3 inflammasomes, with antioxidant and anti-inflammatory properties. Glibenclamide effectively blocks caspase-1 activation and 1L-1*β* secretion *via* TLR4 signaling (Lamkanfi et al., 2009). As NAFLD prevalence is very closely associated with obesity and diabetes, glibenclamide has been tried to treat with NAFLD. In a clinical trial (NCT00494312), glibenclamide was found to have similar effects to pioglitazone in patients with poorly controlled type 2 diabetes, with a good safety profile in liver during long-term use. In a rat model study, glibenclamide attenuated HFD and streptozotocin-induced NAFLD disease, and inhibited hepatic expression of NLRP3 inflammasome components. The hepatoprotective effects of glibenclamide were accompanied by reduced levels of triglycerides, glucose, and cholesterol; mediated by decreased hepatic apoptosis, DNA damage, and inflammatory cytokines; and managed by improving the insulin signaling pathway and antioxidant status (Dwivedi and Jena, 2020). Glibenclamide has shown hepatoprotective ability, and more clinical studies focused on its efficacy for the treatment of NAFLD are warranted.

#### Parthenolide

Parthenolide is a sesquiterpene lactone isolated from *Tanacetum parthenium* (L.) Sch.Bip. (feverfew) that exhibits a broad range of anti-inflammatory effects. As a key ingredient in botanical drugs, parthenolide has been extensively used for the treatment of numerous inflammatory-related diseases. Parthenolide can inhibit caspase-1 activation by catalyzing alkylation of caspase-1 cysteine residues and also directly target the ATP enzymatic activity of NLRP3 by cysteine modification. In a rat model of NAFLD, parthenolide exhibited a hepatoprotective role, as well as anti-inflammatory and anti-oxidative effects. HFD caused significant weight gain and increased liver triglyceride content as well as alteration in alanine aminotransferase (ALT) and aspartate aminotransferase (AST) activities, which were attenuated by administration of parthenolide (Bahabadi et al., 2017). Parthenolide is a potential NAFLD prevention candidate; however, more evidence is needed to support this hypothesis.

#### Bay11-7082

Bay11-7082 is a phenyl vinyl sulfone compound, which inhibits the NF-κB pathway by blocking IKK-*β* kinase activity through alkylation of essential nucleophilic residues. Bay11-7082 suppresses ASC pyroptosome organization and NLRP3 inflammasome formation by alkylating cysteine residues on the ATPase domain of NLRP3 (Jiang et al., 2017). Yu Y. et al. (2019) demonstrated that pretreatment with BAY11-7082 could attenuate mtDNA release from hepatocytes in HFD-fed mice and suppress the induction of TNF-*α* and IL-6 expression in Kupffer cells. Bay11-7082 may be useful for NAFLD treatment; however, more supportive evidence is needed.

### Inhibitors of NLRP3 Inflammasome Constituents and Products

#### Emricasan

Emricasan (IDN-6556) is an irreversible pan-caspase inhibitor involved in caspase-mediated inflammation, necrosis, and apoptosis. Emricasan can be administered orally and is retained for relatively long periods in the liver, where it inhibits hepatocyte apoptosis by suppressing apoptotic-related caspases. This inhibition of cell damage and control of pro-inflammatory release may interrupt and prevent hepatic stellate cell activation, fibrogenesis, and various features of the inflammatory milieu (Barreyro et al., 2015). One randomized controlled trial showed that alanine transaminase and other biomarkers were decreased, and aminotransferases increased in patients with NAFLD after treatment with emricasan for 28 days (Shiffman et al., 2019). In a phase III clinical trial, emricasan could not reduce severe portal hypertension and pressure in patients with NASH and related cirrhosis; however, some patients with higher baseline hepatic venous pressure gradient (HVPG) showed a modest improvement in this index after treatment with different doses of emricasan (NCT02960204) (Garcia-Tsao et al., 2020). In another phase II clinical trial, emricasan could not ameliorate liver fibrosis histology in NASH-related patients, and treatment led to worsened ballooning fibrosis severity (NCT02686762) (Harrison et al., 2020). Furthermore, a recent clinical trial found that emricasan was safe, but did not reduce decompensation events or improve liver function in patients with NASH-related conditions, along with an extensive period of decompensated cirrhosis (NCT03205345) (Frenette et al., 2021). These findings suggest that the therapeutic effect of emricasan in NAFLD warrants further exploration.

#### *β*-Hydroxybutyrate

*β*-Hydroxybutyrate (BHB), a ketone-related metabolite, functions as an NLRP3 inflammasome blocker by inhibiting K^+^ efflux, ASC speck oligomerization, and inflammasome formation (Youm et al., 2015). BHB can effectively block NLRP3 inflammasome activation and lower IL-1ß and IL-18 production. Analysis of clinical data showed that reduced BHB was negatively correlated with hepatic fat accumulation in individuals with obesity-related NAFLD (Mey et al., 2020). Hence, BHB may block NAFLD pathologies as a surrogate measure of hepatic ketogenesis (Luukkonen et al., 2021); however, no pharmacological or dietary treatment experiments using BHB in cells or animal models have been reported.

#### Andrographolide

Andrographolide is a botanical compound, extracted from *Andrographis paniculata* (Burm.f.) Nees, and acts an NF-κB inhibitor, indicating its potential anti-inflammatory ability. Cabrera et al. (2017) showed that andrographolide could ameliorate inflammation and fibrogenesis in experimental NASH and that it clearly reduced ASC and NLPR3 expression to suppress inflammasome activation. Andrographolide derivatives are also involved in cellular and rodent models of NAFLD. Selected isoandrographolide derivatives showed hepatoprotective effects in steatotic HepG2 cells and HFD-fed rats with NAFLD. A diterpenoid, 14-deoxy-11,12-didehydroandrographolide, which is a bioactive phytonutrient, can suppress hepatic protein levels of caspase-1 and NLRP3, and reduce liver inflammation and injury in mouse models induced by both HFD and high-cholesterol diet (Liu Y. T. et al., 2020). Andrographolide and its derivatives also show therapeutic potential for NAFLD treatment and evaluation of their safety, and therapeutic effects could be evaluated in future clinical studies.

#### Compound C

Compound C, a pyrazolopyrimidine, is widely used as a cell-permeable ATP-competitive inhibitor of AMP-activated protein kinase (AMPK). Compound C significantly reduces steatohepatitis incidence and can protect mice from HFD-induced obesity and NAFLD. Compound C down-regulates the expression of NLRP3 inflammasome components and pro-inflammatory markers, and has been defined as an attractive suppressor of NLRP3 inflammasomes, which is a promising therapeutic agent for treatment of NAFLD and related metabolic disorders (Wang et al., 2019). More studies focused on this compound are warranted.

#### Necrosulfonamide

Necrosulfonamide was identified as a direct chemical inhibitor of GSDMD, the pyroptotic pore-forming protein, and binds directly to GSDMD to inhibit pyroptosis. Necrosulfonamide suppresses p30-GSDMD oligomerization to control pore formation. Majdi et al. found that necrosulfonamide can prevent membrane pore formation and attenuate the process of pyroptosis in mice with sepsis (Majdi et al., 2020). To mediate its role in inhibiting pyroptotic cell death, necrosulfonamide also blocked IL-1*β* release from both human and murine macrophages/monocytes. In another study, treatment with necrosulfonamide reduced lipid content in HepG2 cells and blocked lipogenesis (Saeed et al., 2019). Further animal studies should be conducted to confirm the role of necrosulfonamide in NAFLD.

#### SGM-1019

SGM-1019, a new small-molecule that specifically targets the P2RX7 receptor, was developed as an inhibitor of the NLRP3 inflammasome for treatment of NASH. P2RX7 belongs to the ligand ATP-gated P2X class of ionotropic receptors that are activated and driven by the high concentrations of extracellular ATP. This activation triggers cytoplasmic ion transport (K^+^ efflux), which is considered the major driver of NLRP3 inflammasome activation (Di Virgilio et al., 2017). In a primate model of liver fibrosis induced by a chemical complex, SGM-1019 treatment could improve NASH pathological processes and protect cynomolgus from liver fibrosis and inflammation (Baeza-Raja et al., 2020). In Kupffer cells and monocytes, SGM-1019 blocks IL-1*β* production, and it also reduces caspase 3/7 activity in hepatocyte, as well as suppressing hepatic stellate cell pro-collagen secretion. A phase II clinical trial to assess SGM-1019 (NCT03676231) has been conducted; however, the study was terminated early because an undefined safety event occurred after only nine patients have been recruited (Alharthi et al., 2020).

### Pharmacological Chemical Agents Associated With NLRP3 Inflammasome Inhibition

#### Liraglutide

Liraglutide, a glucagon like peptide-1 (GLP-1) analog, has recently emerged as an effective first-line treatment for T2DM and has been found to reduce hepatic steatosis. In one mouse model, liraglutide improved HFD-induced NAFLD and alleviated hepatitis in mice by suppressing hepatic NLRP3 inflammasome activation (Zhu et al., 2018). A study by Yu et al. also indicated that liraglutide can ameliorate NAFLD through controlling the activation of the NLRP3 inflammasome and pyroptosis to regulate mitophagy (Yu X. et al., 2019). Liraglutide can also modulate gut microbiota and reduce NAFLD in obese mice. There is also clinical evidence that GLP-1 can improve liver function and histological resolution of NAFLD. In a double-blind, multi-center, placebo-controlled, and randomized phase II study (NCT01237119), liraglutide showed good safety and tolerance, and improved NASH after 48 weeks of treatments (Armstrong et al., 2016). In another randomized trial, treatment with liraglutide led to increased weight loss, reduced body fat mass, and improved blood glucose control in patients with T2DM and NAFLD. Furthermore, the improvements in waist circumference, fat mass, and weight contributed favorably to hepatic function (Feng et al., 2019). These results indicated that liraglutide can be considered for early treatment of patients with T2DM and NAFLD as preemptive therapy.

#### Exenatide

Exenatide is a GLP-1 receptor agonist that enhances insulin secretion and is used as a drug for T2DM treatment. Exenatide has been proven to attenuate NAFLD in both *in vivo* and *in vitro* studies. Mitochondrial tri-carboxylic acid cycle flux is ameliorated, and insulin resistance, steatosis, and hepatocyte lipotoxicity significantly decreased by exenatide treatment. Furthermore, exenatide can alleviate the injury caused by oxidative stress and control NLRP3 inflammasome activation through regulating the pathway of mitophagy and autophagy in the liver, and has a protective role in the liver of mice with NAFLD and diabetes (Shao et al., 2018). In a clinical trial, exenatide reduced body weight, controlled blood glucose, and improved liver enzyme activity, thus alleviating the pathological features of NAFLD in patients with T2DM (Fan et al., 2013). In a randomized controlled clinical trial, exenatide clearly reduced liver fat content in patients with drug-naïve T2DM and NAFLD, and led to greater reductions of body weight, visceral fat, and liver enzymes than insulin glargine (Liu L. et al., 2020). Several studies have shown that exenatide has a predominant role in reducing ALT levels, thereby promoting weight loss, inhibiting inflammation to some extent, and preventing deterioration of steatosis (Ghazanfar et al., 2021). Exenatide may be incorporated into streamlined individualized therapy for NAFLD.

#### Ocaliva

Ocaliva (obeticholic acid) is a ligand of FXR which maintains liver homeostasis and plays an important role in cellular function and mitochondrial integrity. Levels of hepatic FXR are negatively associated with the degree of NLRP3 inflammasome activation in NAFLD caused by either hepatitis B virus or liver failure in patients and liver injury in mice. FXR inhibits ER stress-induced NLRP3 inflammasome activation in hepatocytes and downregulated NLRP3 and TXNIP expression *via* the PERK–CHOP pathway (Han C. Y. et al., 2018). Ligand-induced induction of FXR activation in the liver assists in defense against metabolic disorders. Indeed, ocaliva has been applied for NAFLD treatment in several clinical trials (Adorini et al., 2012). In a phase III study (NCT02548351), ocaliva clearly ameliorated fibrosis and improved pathological symptoms in patients with NASH and had predicted clinical benefit. More clinical studies are warranted to confirm the effective role of ocaliva for NAFLD treatment.

#### Resveratrol

As a natural polyphenol with phytoalexin function, resveratrol (3,5,4′-trihydroxystilbene) can arrest the progression of some infections in plants. Resveratrol has positive effects on lipid accumulation and lipid profile, as well as improving insulin sensitivity, and has strong anti-inflammatory and antioxidant properties, making it a potential hepatoprotective agent for use in NAFLD therapy. Administration of resveratrol significantly improved blood glucose control and TG content in the serum and liver. Furthermore, resveratrol treatment ameliorates hepatic metaflammation and fatty liver, accompanied by alterations in NLRP3 inflammasomes, and reduces levels of pro-inflammatory markers (Yang and Lim, 2014); however, in a meta-analysis of resveratrol supplementation in patients with NAFLD, the analysis of a total of 302 patients with NAFLD in seven randomized clinical trials found that current evidence was insufficient to demonstrate the efficacy of resveratrol for control and treatment of NAFLD (Jakubczyk et al., 2020). Further research in this area is needed.

#### Verapamil

Verapamil is a calcium channel blocker that improves insulin resistance and hyperglycemia in the patients with metabolic syndrome. Indeed, numerous clinical observations have provided evidence of favorable effects of calcium channel blockers against obesity-related metabolic pathologies in both human and animal models (Park and Lee, 2014). Verapamil has been used to treat high blood pressure and to control angina. One study showed that verapamil can reduce liver inflammation and improve metabolic homeostasis in patients with NAFLD. Another investigation showed that treatment with verapamil can attenuate HFD-induced NAFLD progression. The mechanism underlying the benefits of verapamil relates to its correlation with functional inhibition of the TXNIP/NARP3 pathway, which leads to remarkably reduced expression of the pro-inflammatory cytokines, IL-18 and IL-1*β* (Zhou et al., 2018). Hence, the observed results demonstrate that verapamil is a potential treatment for HFD-induced NAFLD.

#### Vitamin D

Vitamin D is a secosteroid hormone with important effects on liver health, as it plays a fundamental role in mineral metabolism. Epidemiological research indicates that patients with NAFLD are more likely to have vitamin D deficiency than the general population and that circulating levels of vitamin D are closely related to the extent of fibrotic progression in patients with NAFLD. Zhang et al. demonstrated that vitamin D can attenuate HFD-induced liver injury by inhibiting pyroptosis and altering gut microbiota in a rat model. Furthermore, vitamin D attenuates HFD-induced hepatic injury *in vivo* and impairment of liver cell viability *in vitro*, as well as inhibiting lipid accumulation, NLRP3 inflammasome activation, and pyroptosis *in vitro* and *in vivo* (Zhang et al., 2021); however, findings regarding the potential benefits of vitamin D for patients with NAFLD in several clinical trials have been inconsistent. The differences among studies may be attributable to variation in the degree of patient liver injuries, comorbidities, and concomitant medications. Findings to date mostly support a beneficial function for vitamin D supplementation, mainly in younger individuals with moderate-to-mild liver damage and shorter term disease duration (Barchetta et al., 2020).

#### Nicotinamide Adenine Dinucleotide

Nicotinamide adenine dinucleotide (NAD) performs an important function in energy homeostasis and cellular metabolism. Levels of hepatic NAD are significantly decreased, particularly in aging humans and mice, suggesting that NAD substrate supplementation could be a potential therapeutic approach for the prevention and treatment of NAFLD. NAD deficiency also decreases the oxidation of fatty acids, promoting steatosis. Oral administration of the natural NAD precursor, nicotinamide riboside, can strongly improve the patient NAFLD phenotypes caused by NAD deficiency alone or HFD; such typical NAFLD phenotypes includes reduced pro-inflammatory factor release, inhibition of monocyte infiltration, suppression of Kupffer cell accumulation, NLRP3 inflammasome inactivation, and decreased hepatic fibrosis (Zhou et al., 2016). Overall, boosting NAD concentrations can be therapeutic in NAFLD.

#### Allopurinol

Allopurinol is an inhibitor of xanthine oxidoreductase and inhibits uric acid generation, which is a metabolite of fructose metabolism and may have a direct role in NAFLD progression and stimulate NLRP3 activation. Khalaf et al. (2019) showed that allopurinol can ameliorate high fructose-induced hepatic steatosis through modification of hepatic lipid metabolism and inflammation. Furthermore, Wang et al. reported that allopurinol can significantly inhibit TXNIP overexpression and NLRP3 inflammasome activation, peroxisome proliferation-activated receptor *α* (PPAR*α*) downregulation, sterol regulatory element-binding protein upregulation, and fatty acid synthase overexpression in diabetic rat liver. Downregulation of hepatic TXNIP by allopurinol can contribute to inhibition of NLRP3 inflammasome stimulation under hyperglycemic conditions, thereby reducing liver inflammation and lipid accumulation (Wang et al., 2013). In addition, allopurinol has a hepatoprotective effect *via* miR200a, which inhibits the TXNIP/NLRP3 inflammasome (Ding et al., 2018). These studies indicate that allopurinol may have therapeutic implications for the prevention of fructose-induced NAFLD.

#### Auranofin

Auranofin is a medicine that has been used to treat rheumatoid arthritis for many years and is reported to have potential effects in diverse diseases. Auranofin has recently been considered as a therapeutic agent for the management of metabolic diseases, particularly treatment of NAFLD by immunomodulation. Hwangbo et al. (2020) demonstrated that auranofin attenuates NAFLD by inhibiting hepatic inflammation caused by NLRP3 inflammasome activation and suppresses lipid accumulation both *in vitro* and *in vivo*. Auranofin is a potential candidate for relieving and improving the symptoms of NAFLD; however, more studies are required to prove its efficacy.

#### Praliciguat

Praliciguat is a soluble guanylate cyclase stimulator which can catalyze cyclic guanosine-3′5′-monophosphate formation by conversion of guanosine triphosphate. Praliciguat is an important cell signaling second messenger with a key role in tissue homeostasis. In a mouse model of NASH, praliciguat inhibited inflammation and fibrosis, and suppressed fibrotic stellate cell transformation (Hall et al., 2019). Furthermore, praliciguat exhibited anti-inflammatory and anti-fibrotic effects in a choline-deficient l-amino acid-defined HFD-induced NASH mouse model. Praliciguat can suppress hepatic levels in NLRP3 inflammasome components, including IL-1*β*, NLRP3, caspase-1, and ASC, and exerts anti-inflammatory function in the liver *via* a vasodilator-stimulated phosphoprotein/NF-κB/NLRP3 circuit (Flores-Costa et al., 2020). Praliciguat can also reduce levels of the active form of cleaved caspase-1 after NLRP3 inflammasome activation, independent of pannexin-1 activity. Together, these studies indicated that praliciguat has a potential therapeutic value in preventing NASH progression.

### Botanical Drugs Associated With NLRP3 Inflammasome Inhibition

#### Berberine

Berberine is a bioactive alkaloid that can be isolated from various botanical drugs including *Coptis chinensis* Franch. and *Hydrastis canadensis* L., which has a variety of pharmacological activities, including antimicrobial, antidiabetic, and anticancer (Lu et al., 2022). Some preliminary clinical reports have confirmed that berberine has an important role in improving hepatic steatosis as a short-term supplement. In an animal study, berberine clearly suppressed NLRP3 expression, and caspase-1 and GSDMD-N activation (Mai et al., 2020). The inhibition of NLRP3 inflammasomes and pyroptosis in NASH by berberine occur *via* the ROS/TXNIP axis. Six randomized clinical trials were evaluated in a meta-analysis, where analysis of data from 501 patients demonstrated and confirmed that berberine has positive effects on insulin resistance, lipid profiles, and hepatosteatosis markers in patients with NAFLD (Wei et al., 2016). Overall, berberine has a potential as an agent for NAFLD treatment, despite its lack of good oral bioavailability, which requires improvement by pharmaceutical modification techniques in the future.

#### Silybin

Silybin (silymarin) is a powerful antioxidant agent extracted from *Silybum marianum* (L.) Gaertn. (Milk thistle), which has a specific tropism for liver (Wang X. et al., 2020). As a traditional Chinese medicine and hepatoprotective agent, silybin has been used for NAFLD treatment worldwide. Silybin can ameliorate hepatic lipid accumulation and modulate global metabolism in many NAFLD mouse models (Ou et al., 2018; Sun R. et al., 2020). Furthermore, silybin can inhibit ER stress and NLRP3 inflammasomes through the NAD/sirtuin2 pathway in mice with NAFLD (Zhang et al., 2018). Moreover, silybin can reduce TXNIP expression, caspase-1 cleavage, and cytokine IL-1*β* release. The protective effects of silybin on the liver are exerted through regulation of NF-κB signaling and NLRP3 inflammasome activation; however, a meta-analysis of randomized controlled trials indicated that intake of silybin was closely related to markedly reduced circulating γ-glutamyltransferase levels. Nevertheless, administration of silybin did not have significant effects on AST and ALT levels (Derakhshandeh-Rishehri et al., 2020). Silybin may reduce fibrosis, but this remains to be confirmed.

#### Glycyrrhizin

Glycyrrhizin is extracted from *Glycyrrhiza glabra* L. (liquorice), a traditional Chinese medicine, and has shown potential hepatoprotective effects in preclinical stage human and animal model studies (Li M. et al., 2021). In a mouse model, glycyrrhizin regulated insulin sensitivity, lipid profiles, and glucose homeostasis to reduce NAFLD progression (Sun et al., 2017). In another mouse model, glycyrrhizin markedly ameliorated hepatosteatosis and fibrosis induced by an MCD diet and suppressed NLRP3 inflammasome activation and inflammation, demonstrating that glycyrrhizin can alleviate NASH *via* modulating bile acids and meta-inflammation (Yan et al., 2018). Furthermore, a clinical study showed that glycyrrhizin tablets exhibit protective effects in the treatment of children with NAFLD (Li Y. W. et al., 2017), indicating that glycyrrhizin could be a therapeutic option for NAFLD treatment. More clinical studies on this medicine are warranted.

#### Baicalin

Baicalin, a flavonoid glycoside that originates from *Scutellaria baicalensis* Georgi root tissue, has strong antioxidant reaction activity, and has been known for many years to exert anti-inflammatory effects in liver diseases (Yang et al., 2021). Baicalin is also a Chinese botanical drug used to treat NASH. One animal study showed that baicalin can attenuate NASH by suppressing important regulators of inflammation, fibrosis, and lipid metabolism in mice. In a cell culture experiment, baicalin could alleviate cytotoxicity induced by palmitic acid in hepatocytes by suppressing NLRP3 inflammasome activation and ER stress (Zhang et al., 2017). In another NASH cell model, baicalin attenuated hepatic injury by inhibiting NLRP3 inflammasome-dependent cell pyroptosis mediated by GSDMD (Huang et al., 2019). These investigations indicate a probable application for baicalin in NAFLD treatment and the potential mechanism underlying baicalin-mediated anti-inflammation.

#### *Lycium barbarum* Polysaccharides

*Lycium barbarum* polysaccharides (LBP), which are composed of a fibrous-like proteoglycan, are the main bioactive components of the traditional medicinal herb, *Lycium barbarum* L*.* (Chinese wolfberry), and have a variety of pharmacological functions, including immunomodulatory and antioxidant activities (Liu et al., 2017; Gao et al., 2021). In a rat model of NASH, administration of LBP for the treatment of steatosis exhibited therapeutic efficiency, reducing hepatic fibrosis and inflammation (Jia et al., 2016). The effect of LBP in hepatoprotection is closely associated with attenuation of TXNIP/NLRP3 and reduced NF-κB activity. The inhibition of NLRP3 inflammasomes and NF-κB activation by LBP treatment partially assisted in the alleviation of hepatic injury during NASH progression (Xiao et al., 2018). Hence, LBP treatment is a potential strategy for NASH prevention.

#### Salvianolic Acid A

Salvianolic acid A (SalA), a natural polyphenol compound, is extracted from *Salvia miltiorrhiza* Bunge and has various potential pharmacological protective roles in NAFLD (Shao et al., 2021). SalA administration clearly attenuated obesity and liver injury induced by a HFD in a mouse model, and significantly diminished lipid accumulation in mouse livers (Li et al., 2020). SalA can reverse TXNIP/NLRP3 pathway activation, fatty acid synthase upregulation, and nuclear translocation of carbohydrate response element-binding protein (ChREBP) caused by HFD or palmitic acid (Ding et al., 2016). Hence, SalA ameliorates HFD-induced NAFLD by regulating the TXNIP/NLRP3 and TXNIP/ChREBP pathways in rats and is a potential candidate for NAFLD treatment, particularly the underlying pathological condition, lipotoxicity.

#### Naringenin

Naringenin is a flavonoid naturally abundant in citrus fruits, especially *Citrus × aurantium* L. (grapefruit), which can prevent liver damage because of its anti-inflammatory, anti-fibrotic, antioxidant, and anticancer properties (Bhia et al., 2021). Administration of naringenin has been tested in various liver damage models induced by hepatic injury agents *in vivo* and *in vitro*, which demonstrated the beneficial effects of naringenin for NAFLD treatment by regulating lipid metabolism, including modulating the oxidation and deposition, and synthesis of lipids and cholesterol. Naringenin also attenuates NLRP3 and IL-1*β* expression, and inhibits NAFLD development by inhibiting the NLRP3/NF-κB signaling pathway in both hepatocytes and Kupffer cells (Wang Q. et al., 2020). Hence, naringenin is considered a potential candidate for the treatment of liver diseases.

#### Salidroside

Salidroside, a phenylpropanoid glycoside compound, is the main active ingredient of *Rhodiola rosea* L. and is proven to have good biological activity against some metabolic diseases, and protected against NAFLD by improving hepatic lipid metabolism in many animal experiments (Wu et al., 2020). Salidroside can suppress hepatic oxidative stress, TXNIP expression, and NLRP3 inflammasome activation in the liver. Furthermore, salidroside improves HFD-induced NASH *via* regulation of the oxidative stress and AMPK-dependent TXNIP/NLRP3 pathways. Overall, there is evidence that salidroside exhibits therapeutic action against NAFLD and warrants further attention.

#### Ginseng Saponin

Ginseng saponin is the main active ingredient of *Panax notoginseng* (Burkill) F. H. Chen contains several ginsenosides, and has various therapeutic and pharmacological features. The administration of ginseng saponin extract clearly regulates the remission of hepatic steatosis, inflammation, and fibrosis induced by HFD (Choi et al., 2019). In particular, saponin extract contains significantly increased amounts of ginsenosides, including Rh1 and Rg2, which cause a blockade of steatosis in hepatocytes, inhibition of fibrosis in hepatic stellate cells, and promote mitophagy in Kupffer cells. Furthermore, ginseng saponin enriched for Rh1 and Rg2 can ameliorate NAFLD by inhibiting NLRP3 inflammasome activation through promotion of mitophagy (Wang F. et al., 2021). Thus, administration of ginseng saponin is likely advisable for NAFLD prevention.

#### Sweroside

Sweroside, an iridoid, is an active component of traditional Chinese medicine *Swertia pseudochinensis* H. Hara. Clinical studies have shown that oral delivery of sweroside significantly prevented chemical component-induced liver injury and fibrosis in rats (Yang Q. et al., 2020). Sweroside may also ameliorate HFD-induced NAFLD *via* regulation of lipid metabolism and inflammatory responses (Yang G. et al., 2020). Alleviation of NASH symptoms by sweroside is mediated by its protective function in inhibiting hepatic NLRP3 inflammasome activation, accompanied by decreased hepatic IL-1*β* and caspase-1 levels (Yang Q. et al., 2020). Sweroside can block the initiation of hepatic mtDNA synthesis, which contributes to suppressing NLRP3 inflammasome activation at source. Furthermore, targeting of the NLRP3 inflammasome by sweroside can provide benefit and assist in NASH symptom improvement, indicating its therapeutic potential for NASH regulation.

#### 4-Acetylantroquinonol B

4-Acetylantroquinonol B (4-AAQB) is a natural ubiquinone derivative extracted from the mycelia of *Antrodia cinnamomea* and is used as traditional medicine in Taiwan (Li and Chiang, 2019). 4-AAQB treatment led to significantly decreased ALT and AST levels in MCD diet-induced NAFLD mice. Moreover, 4-AAQB inhibited the activation of NLRP3 inflammasomes, responses of inflammatory cytokines, and ER stress in various *in vitro* and *in vivo* models. Furthermore, Sirtuin 1 and Nrf2 signaling pathways were increased and protected the liver from injury (Yen et al., 2021). Hence, current data indicate that treatment with 4-AAQB represents an accessible therapeutic strategy for NAFLD control.

#### Magnolol

Magnolol, the major dynamic compound in *Magnolia officinalis* Rehder and E. H. Wilson, has extensive biological activities (Lin et al., 2021). In a rat model, magnolol showed a protective effect against hepatic steatosis, and reduced plasma IL-1*β* and protein expression of NLRP3/ASC/caspase-1 in hepatocytes. Furthermore, magnolol suppressed NLRP3 inflammasome activation and hepatic steatosis *via* promotion of autophagy and regulation of heme oxygenase-1 signaling, to ameliorate inflammatory responses and oxidative stress (Kuo et al., 2020). These findings imply that magnolol represents a promising approach for NAFLD management.

#### Cannabidiol

Cannabidiol is a non-psychoactive component abundant in *Cannabis sativa* L. (marijuana), which preliminarily studied has shown to have a protective role aganist hepatic steatosis (Peng et al., 2022). The administration of cannabidiol may help to ameliorate symptoms of NAFLD induced by HFD and can significantly inhibit the nuclear translocation of NF-κBp65 and NLRP3 inflammasome activation, helping to reduce the expression of inflammatory factors *in vivo* and *in vitro*. Furthermore, the anti-inflammatory function of cannabidiol in the liver is mediated by deactivation of NF-κB to control NLRP3 inflammasome activation in macrophages (Huang et al., 2019), which is a potential method for the treatment of NAFLD.

#### Dieckol

Dieckol has various beneficial functions, including antioxidant, anti-inflammatory, and anti-adipogenic effects (Rajan et al., 2021). Dieckol-enriched extracts from *Laminaria japonica* can decrease hepatic steatosis by stimulating hepatic fatty acid *β*-oxidation. In an HFD-induced animal model, administration of dieckol-enriched extraction from *Ecklonia cava* extracts could attenuate NAFLD syndrome. Furthermore, HFD-induced NLRP3 inflammasome expression, including genes encoding NLRP3, ASC, and caspase-1, was decreased in response to dieckol. Moreover, the number of pyroptotic cells induced by a HFD was also reduced by the administration of dieckol (Oh et al., 2021). Hence, dieckol inhibits lipid accumulation in the liver by increasing lipolysis and decreasing lipogenesis, which may be beneficial for the treatment of NAFLD.

#### Apigenin

Apigenin is a natural bioflavonoid present in various vegetables, herbs, spices, and fruits, and particularly in *Citrus × aurantium* L. (grapefruit) (Kashyap et al., 2021). Apigenin can improve and ameliorate HFD-induced NAFLD syndrome in a mouse model by decreasing inflammation and hepatic lipid accumulation. The protective function of apigenin may be partially attributable to the regulation of xanthine oxidase activity, which subsequently influences NLRP3 inflammasome activation, and IL-1*β* and IL-18 cytokines release. Therefore, apigenin is a potential therapeutic candidate for use in the prevention of NAFLD (Lv et al., 2019).

#### Antrodia cinnamomea

*Antrodia cinnamomea*, an indigenous medicine, is widely used by Taiwanese aboriginal tribes. Numerous studies have revealed various biological activities of *Antrodia cinnamomea*, including hepatoprotective, anti-hyperlipidemic, and anti-inflammatory effects (Chyau et al., 2020). Yen et al. demonstrated that *Antrodia cinnamomea* clearly suppresses NLRP3 inflammasome activation *in vivo* and *in vitro*. In addition, *Antrodia cinnamomea* reduced the severity of acute hepatitis induced by an MCD diet, attenuated expression of oxidative stress-related markers, and improved inflammatory responses underlying fatty liver, as well as restoring and promoting autophagy (Yen et al., 2020). Hence, *Antrodia cinnamomea* is an effective medicine for the management and regulation of NAFLD progression.

#### Benzyl Isothiocyanate

Benzyl isothiocyanate, a phytochemical organosulfur component extracted from glucosinolates by hydrolysis, was originally found in cruciferous vegetables particularly in *Lepidium sativum* L. that exhibits anti-inflammatory effects (Dinh et al., 2021). Benzyl isothiocyanate attenuates fatty liver in HFD-induced obese mice, and could also ameliorate cholesterol- and cholic acid diet-induced NASH by suppressing activated NLRP3 inflammasome stimulation by cholesterol crystals in Kupffer cells (Chen et al., 2020). Therefore, benzyl isothiocyanate is a potential chemical preventive agent against NASH development primarily induced by the diet.

#### TSG

TSG (2,3,5,4′-tetrahydroxy-stilbene-2-O-*β*-D-glucoside), a natural stilbene, mainly distributed in *Reynoutria multiflora* (Thunb.) Moldenke, exhibits strong ability to regulate hepatic lipid metabolism and is particularly effective in lowering hepatic triglyceride levels, thereby preventing HFD-induced NAFLD progression (Li H. et al., 2017). TSG is also effective in preventing triglyceride accumulation in hepatic cells and significantly reduced NAFLD biochemical indexes. Moreover, TSG can reduce the levels of NLRP3, caspase-1, and ASC, as well as regulate functional gut microbiota in mice with NAFLD induced by an MCD diet. As NAFLD therapy, TSG could be selected as a lead promising agent that may contribute to disease prevention (Han M. et al., 2018).

#### Sulforaphane

Sulforaphane is a natural product enriched in *Brassica oleracea* L. that can reduce the accumulation of lipids *in vivo* and *vitro* (Milito et al., 2019; Li J. et al., 2021). Two randomized clinical trials have shown that intake of broccoli can reduce inflammatory markers and low-density lipoprotein cholesterol. A study by Yang et al. showed that oral administration of sulforaphane could prevent the development of NAFLD induced by HFD, which was regulated by hepatic NLRP3 inflammasome expression in a mouse model (Yang et al., 2016). The effects of sulforaphane on NLRP3 inflammasome are mediated through the AMP-activated protein kinase-autophagy axis. Sulforaphane administration alleviates HFD-induced hepatic steatosis symptoms in NAFLD in a mouse model (Wu et al., 2021). Sulforaphane is a promising intervention to treat or relieve NAFLD, and the clinical study is urgently warranted.

#### Chaihu Shugan San

Chaihu Shugan San is a popular Chinese botanical drug used in numerous traditional formulas; consists of *Citrus × aurantium* L*.*, *Bupleurum falcatum* L., *Conioselinum anthriscoides* “Chuanxiong,” *Paeonia lactiflora* Pall., *Cyperus rotundus* L*.*, and *Glycyrrhiza glabra* L.; and has been widely applied clinically for many years in China (Zhu et al., 2021). Chaihu Shugan San is beneficial for the treatment of various chronic diseases. A study by Liang et al. demonstrated that Chaihu Shugan San decoction can modulate intestinal microflora dysbiosis, reduce fat accumulation, and alleviate chronic inflammation by inhibiting the NLRP3 inflammasome pathway in rats with NAFLD (Liang et al., 2018). Chaihu Shugan San is a potentially complementary medicine that has demonstrated functionality in protecting the liver from injury, treating moderate hepatic inflammation reaction, and alleviating NASH progression.

#### Fufang Zhenzhu Tiaozhi

Fufang Zhenzhu Tiaozhi (FTZ) consists of *Coptis chinensis* Franch., *Ligustrum lucidum* W.T.Aiton, *Salvia miltiorrhiza* Bunge, *Eucommia ulmoides* Oliv., *Panax notoginseng* (Burkill) F.H.Chen, *Cirsium japonicum* DC., *Citrus medica* L*.*, and *Atractylodes macrocephala* Koidz., and is a traditional Chinese herbal formula with various pharmacological properties, including anti-hypoglycemic, anti-hypolipidemic, anti-inflammatory, and anticoagulant activities (Wang H. et al., 2021). Therefore, FTZ is mainly used in China for the treatment of metabolic syndrome, and in particular for hyperlipidemia in NAFLD. FTZ formula can not only significantly attenuate liver steatosis and fibrogenic phenotype but also reduce NLRP3 inflammasome formation and activation (Chen et al., 2018). Overall, FTZ represents a therapeutic option for the treatment of metabolic syndrome in patients with NAFLD.

#### Gegen Qinlian Decoction

Gegen Qinlian Decoction (GQD) consists of *Pueraria montana var. lobata* (Willd.) Maesen & S. M. Almeida ex Sanjappa & Predeep, *Scutellaria baicalensis* Georgi, *Coptis chinensis* Franch*.*, and *Glycyrrhiza glabra* L.; is a classical, traditional Chinese botanical drug that has been used extensively in clinics in China for hundreds of years; and has definite effects in the treatment of NAFLD (Lu et al., 2021). Furthermore, GQD can block hepatic NLRP3, caspase-1, and ASC expression, further inhibiting NLRP3 inflammasome activation. These studies suggest that GQD can ameliorate hepatic injury and steatosis in NAFLD. The mechanism underlying the protective role of GQD in the liver involves modulation of anti-oxidative factors, regulation of inflammatory cytokine release, and suppression of NLRP3 signaling (Ying et al., 2021). Findings to date support GQD as a potential candidate for NAFLD treatment in the future.

## Concluding Remarks

The inflammatory response caused by NLRP3 inflammasome activation is essential in NAFLD development, and particularly in the process of progression from NAFLD to NASH. To control the high rates of morbidity and mortality in patients with NASH, pharmacotherapies should be applied for those patients with fibrosis. NLRP3 inflammasome-induced cytokines release and pyroptosis processes are the key inflammatory targeting signals in liver disease; therefore, medicines involved in the regulation of the NLRP3 inflammasome represent the primary therapeutic approach for NAFLD management. There have been various discoveries and strategies related to NLRP3 inflammasome targets and blockades. The most effective pharmacological medicines are closely connected to NLRP3 inflammasomes that are characteristics of NAFLD.

In this review, we summarized information regarding numerous medicines related to NLRP3 inflammasomes, including direct NLRP3 inhibitors, inhibitors of the inflammasome constituents, pharmacological chemical medicines, and botanical drugs. These four categories of NLRP3-associated agents have different characteristics. First, NLRP3 inhibitors directly target the NLRP3 receptor itself and have the advantage of specificity. The efficacy and safety of such agents warrant further clinical study. Second, inhibitors of NLRP3 inflammasome constituents mainly target the processes involved in inflammasome activation. This type of inhibitors can strongly influence other molecular signalings more than NLRP3 inflammasome molecules compared with NLRP3 inhibitors, leading to increased uncertainty in clinical investigation. Third, chemical agents associated with NLRP3 inflammasomes function to ameliorate NAFLD, and some of their mechanisms are related to NLRP3 inflammasome blockade. These agents show limited specificity, compared with the other two types of inhibitors discussed earlier; however, they have demonstrated potential to regulate the NLRP3 inflammasome as NAFLD treatment in clinical and animal studies. Fourth, herbal agents associated with the NLRP3 inflammasome can also function in ameliorating NAFLD; these have more complex ingredients, and their underlying mechanism are related to suppress the NLRP3 inflammasome.

To date, only a small proportion of NLRP3-associated agents have undergone preclinical testing in various NAFLD/NASH models. Nevertheless, some have achieved good prospective results in the clinic, including liraglutide, exenatide, glibenclamide, ocaliva, and berberine. Additionally, most of the agents discussed in this review have clinical potential; however, they remain at the animal model stages of research. The effectiveness of these agents should be further investigated in clinical trials, and a risk analysis is required. Moreover, patient-centered cost-benefit and cost-effectiveness data are urgently needed to position these medications for consideration in practical guidelines for the treatment of NAFLD/NASH. There remain considerable prospects and challenges for the use of NLRP3 blockade to treat NAFLD/NASH, and optimization of the therapeutic strategy for NAFLD remains a major challenge. Future research directions should focus on agents with good results in prospective clinical studies and increase the clinical research into use of these agents for the treatment of NAFLD.
